# Trueness and Precision of Three-Dimensional Digitizing Intraoral Devices

**DOI:** 10.1155/2018/5189761

**Published:** 2018-11-26

**Authors:** Hussam Mutwalli, Michael Braian, Deyar Mahmood, Christel Larsson

**Affiliations:** ^1^Department of Prosthodontics, Faculty of Odontology, Malmö University, Smedjegatan 16, 214 21 Malmö, Sweden; ^2^Department of Materials Science and Technology, Faculty of Odontology, Malmö University, Smedjegatan 16, 214 21 Malmö, Sweden

## Abstract

**Aim:**

To measure the trueness and precision under repeatable conditions for different intraoral scanners (IOSs) when scanning fully edentulous arch with multiple implants.

**Materials and Methods:**

Three IOSs and one industrial scanner were used to scan one edentulous master cast containing five implant scan bodies and three spheres. The cast was scanned thirty times with each scanner device. All scans were analyzed in the inspect software, and three-dimensional locations of the implants and the interarch distance between the spheres were measured. The values were compared to measurements made with one coordinate measuring machine (true value). One-way ANOVA was used to calculate the differences between IOSs and in comparison with the true value.

**Results:**

Significant differences were found between all IOSs. For the implant measurements, Trios 3 had the lowest trueness (≤114 *μ*m), followed by Trios 3 mono (≤63 *μ*m) and Itero element (≤−41 *μ*m). Trios had the lowest precision (≤135 *μ*m), followed by Itero element (≤101 *μ*m) and Trios 3 mono (≤100 *μ*m). With regard to the interarch distance measurements, Trios 3 had the lowest trueness (≤68 *μ*m), followed by Trios 3 mono (≤45 *μ*m) and Itero element (≤40 *μ*m). Trios 3 had the lowest precision (≤206 *μ*m), followed by Itero element (≤124 *μ*m) and Trios 3 mono (≤111 *μ*m).

**Conclusion:**

The results from this in vitro study suggest that precision is low for the tested IOS devices when scanning fully edentulous arches with multiple implants.

## 1. Introduction

One of the most recent techniques introduced to dentistry is the ability to digitize the oral cavity and create a three-dimensional virtual model; this device is known as an intraoral scanner (IOS). The first appearance of the IOS was in 1980. Few years later, a Swiss dentist and an Italian electrical engineer developed and introduced CEREC by Sirona Dental Systems in 1987 [1]. Over the past few years, several commercial IOS systems have been introduced to the market. Preferably, the IOS device should have high trueness and high precision. Both trueness and precision describe the accuracy of the specific digital device (ISO 12836:2015) [2]. High trueness means that the device provides a result that is close to or equal to the true dimension of the object being scanned. A device with higher precision has more repeatable and consistent scans or measurements. This is however not always achieved in all scanners at all clinical conditions. Several studies have shown that IOS devices have difficulty in scanning full dental arch or edentulous arch with multiple implants and generating accurate virtual models [3–8]. The sources that could generate errors are scanning software process, oral environment, scanning protocol, and user's experience. The main reason for enhanced errors on longer span scans could be the scanning method found in most IOS devices. The scanners acquire single images that are stitched with other images to produce a virtual 3D model of the object being scanned. The technique is referred to as stitching; software process known as the best fit algorithm, can introduce errors into large scan distance such as the full-arch situation [6, 9, 10]. To achieve a proper stitching, the scanned object needs a suitable structure. Typically, occlusal surfaces on molars and premolars present structures with many anatomical geometries making these areas simpler to stitch compared to edentulous areas or the incisal edge of the mandibular front teeth [11]. The oral environment contains saliva, blood, and artificial reflective surfaces in the oral cavity that can introduce errors during the digitizing process [12]. The scanning protocol refers to the scanning path for digitizing the object. A study reported that an accurate scanning strategy minimizes the inaccuracies in the digital fabrication workflow and creates precise virtual 3D datasets [13]. Therefore, it is crucial to apply the right scanning path in order to obtain a usable virtual 3D model [14]. In addition to the scanning protocol, the learning curve or user's scanning skill has an impact on generating accurate virtual models. Studies report that experienced operators can perform better scans than nonexperienced operators [9, 10]. After scanning, restorations are designed in computer-aided software and then milled or additively manufactured from materials ranging from polymers to monolithic ceramics. In order to control errors in this digital workflow, it is important to study each process in the production.

**Figure 1:**
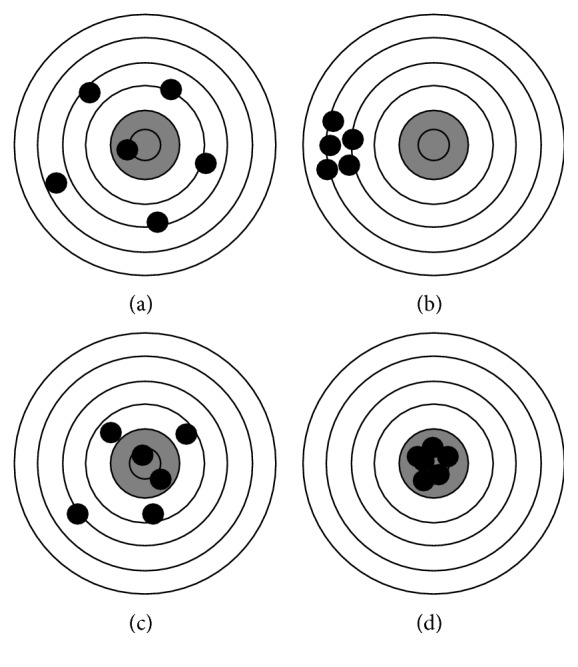
Illustration of the correlation between accuracy and precision. (a) Low accuracy, low precision. (b) Low accuracy, high precision. (c) High accuracy, low precision. (d) High accuracy, high precision.

The current study focused on assessing the trueness and precision of three different intraoral scanners on the scanning edentulous jaw with multiple implants. Two terms are central to understanding metrology: accuracy and precision. Accuracy relates to the closeness of a measured value to a standard or a known (true) value, whereas precision pertains to the closeness of measured values to each other.  Figure 1 illustrates the correlation between these two terms; if the center of the target would be referred to as the true value or the standard, then Figure 1(a) shows a result that has both low accuracy and precision. In contrast, Figure 1(d) illustrates results that are both accurate and precise. When conducting research, it is normal to calculate the standard deviation (SD) and the mean value of a measurement (mean). Relating these two terms to the metrological nomenclature would correlate the mean value as accuracy and the standard deviation to precision. The authors have utilized the recently updated recommendation from the International Organization for Standardization (Dentistry—Digitizing devices for CAD/CAM systems for indirect dental restorations—Test methods for assessing accuracy, ISO 12836:2015) as reference for describing the terms accuracy, trueness, and precision [2]. ISO 12836:2015 uses two terms to describe accuracy; the first is precision and the second is trueness. Trueness is closeness of agreement between the mean obtained from repeated measurements and a true value. If ISO 12836:2015 is used as reference, then all accuracy legends in Figure 1 would change to trueness, and Figure 1(d) would be regarded as accurate and Figure 1(a) as not accurate; meanwhile, Figure 1(b) would be described as having a low trueness; the shooter is precise but not accurate. Precision is the closeness between the independent results of measurement obtained under specific conditions. Precision is divided into two different groups: in the first group, the subject is tested in the same way by the same operator and measuring equipment under the same conditions. This first version of precision tests repeatability. The first version was tested in this current study. According to ISO, the measurement should be repeated thirty times under the same condition to assess the precision under repeatable condition. The precision would be obtained by calculating the standard deviation of the thirty measurements. In the second version of precision, the conditions change. Thus, this part tests reproducibility.

Different methods have been developed by researchers to assess the trueness and precision of IOS devices. Some of these studies have either compared the IOS device to the conventional impression technique or to other IOS devices [6–8, 15, 16]. Almost all of the researchers employed a master cast that has been measured either by tactile computer metric measurements (CMM) or by using an industrial optical scanner to obtain reference data as a virtual 3D file. The master cast is then scanned by the test scanners, obtaining virtual models. The virtual models are measured and compared to the reference date. Some studies employed the best fit alignment method to assess full-arch scans with teeth or implants [4, 7, 8, 16]. The method presents a color map data with threshold colors to visualize and measure differences between two scans. The current study used a different method: here, five cylinders (scan bodies) and three spheres were used as landmarks, making it possible to calculate only eight points in the scan, instead of comparing thousands of points from the scan data. The aim of this present study was to measure the trueness and precision under repeatable condition of different intraoral scanner devices on scanning a fully edentulous arch with multiple implants. The null hypothesis was that there would be no significant differences between the intraoral scanner devices in comparison with the true value (coordinate measuring machine).

## 2. Materials and Methods

### 2.1. Master Model

**Figure 2:**
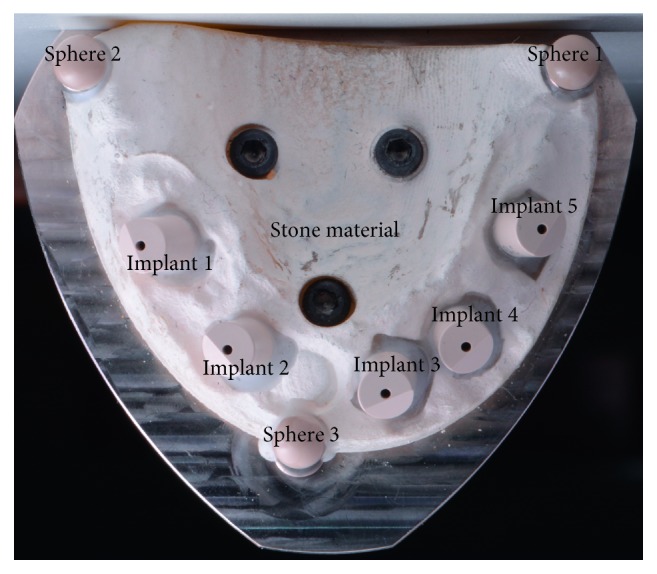
Master model.

A cast of an edentulous maxilla with five implants (Brånemark system Mk IV TiUnite WP 5 × 10 mm). Five implant scan bodies (Elos Accurate Intraoral Brånemark WP 6A-C, Elos Medtech Pinol A/S, Gørløse, Denmark) were connected to the implants. The scan bodies were manufactured from polyether ether ketone (PEEK). The core structure of the model consisted of tungsten metal, and the edentulous areas were made by using dental stone material. The implants were placed in a nonparallel position in order to mimic the clinical situation. The implants were positioned in the area of the lateral incisor, canines, and second premolars, according to positions 1–5, as illustrated in Figure 2. Three spheres were used as fixed reference landmarks; the placement is shown in Figure 2.

### 2.2. Coordinate Measuring Machine (CMM)

**Figure 3:**
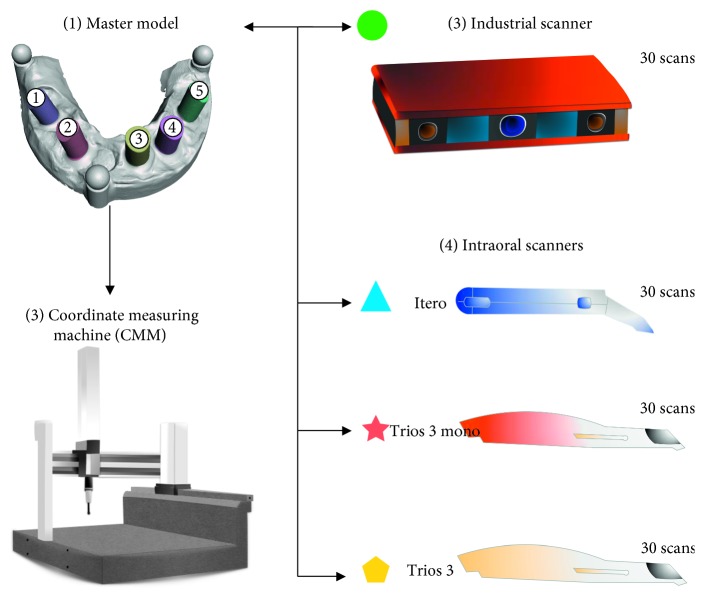
Study workflows. (1) Master model. (2) CMM measured the master model to obtain a true value. (3) Industrial Atos scanner digitized the master model 30 times. (4) Each intraoral scanner device (Trios 3, Trios 3 mono, and Itero) digitized the master model 30 times.

The master model was measured by a certified metrologic center (Elos Medtech Pinol A/S, Gørløse, Denmark) to obtain three-dimensional data of the master model components (Figure 3). A certified industrial coordinate measuring machine, CMM (Carl Zeiss Industrielle Messtechnik GmbH) was used with its corresponding software (Calypso 2015, Service Pack 3, Version 6.0.12). The master model was measured one time with the CMM using a high touch signal probe with a 1 mm ruby sphere (in three dimensions: *x*, *y*, and *z* axes). The accuracy of the CMM was certified by the national entity of accreditation with a maximum permissible error of length measurement of 1.9 ± 3 *μ*m/1,000 *μ*m according to the appropriate standard published by the International Organization for Standardization [17]. The circumference of each scan bodies was measured to define its center point in the *x*, *y*, and *z* axes. Furthermore, the interarch distance between the center of spheres was determined. The CMM measurement was regarded as a true value in the present study.

### 2.3. GOM Optical 3D Metrology

The master model was sent to the manufacturer center (Kulzer GmbH Nordic AB, Helsingborg, Sweden) to scan the master model thirty times with an optical industrial blue light scanner (Atos core, GmbH Optical Metrology, Braunschweig, Germany) (Figure 3). The system technology of this optical scanner is based on emitting different light fringe patterns onto the model, while the reflected lights are recorded by means of two high-resolution video cameras. Atos scanner was used in this study as a reference scanner for comparison with IOS devices.

### 2.4. Intraoral Scanner Systems

Three different IOS systems were evaluated: Trios 3 and Trios 3 mono (3shape, Copenhagen, Denmark) and Itero (Align technology Inc., California, USA) (Table 1). The master model was scanned 30 times with each tested scanner device, resulting in 90 virtual 3D models in total (Figure 3). To reduce the risk of operator bias and different levels of operating experience that could influence the results, it was decided that all scanning procedures would be made by one dentist who was experienced and familiar with the three IOS systems. The calibration of all IOS systems was performed according to the manufacturer's recommendations in order to mimic the clinical situation.

#### 2.4.1. Trios 3 Scanner

The Trios 3 scanner is based on confocal microscopy technology. The system has color software, and powder application is not required. Trios 3 is cabled (pod). The master model was scanned 30 times on the same day with approximately 10 to 15 minutes between each scan. The scanning procedure started first from the buccal surface of the right sphere (sphere 3), moving along the buccal surface toward the left sphere (sphere 1), and returning from the occlusal-palatal side of sphere 1. This technique was called buccal, occlusal, and palatal scanning strategy (BOP).

#### 2.4.2. Trios 3 Mono Scanner

Trios 3 mono has the same features as Trios 3, except for its inability to register color. Trios 3 mono is cabled (pod). Trios 3 mono was utilized to repeatedly scan the master model 30 times on the same day with approximately 10 to 15 minutes between the scans. This was followed by a similar scanning strategy (BOP) to scan the master model.

#### 2.4.3. Itero Element Scanner

The Itero element device is a powder-free system with color scan features. The Itero technology is based on parallel confocal microscopy to capture several images per second. In order to evaluate the repeatability of the Itero device, the master model was scanned 30 times during the same session with approximately 10 to 15 minutes between each scan. The scanning strategy for the Itero group was performed by zigzag movement from sphere 3 to sphere 1 (buccal to occlusal to palatal), all in one direction and without returning to the starting point. This strategy was chosen on account of fact that the Itero scanner tip is larger in width than the other dental scanner; therefore, it was almost impossible to scan the mesial and distal surface properly without rotating the camera tip.

### 2.5. Alignment and Measurement Procedures

**Figure 4:**
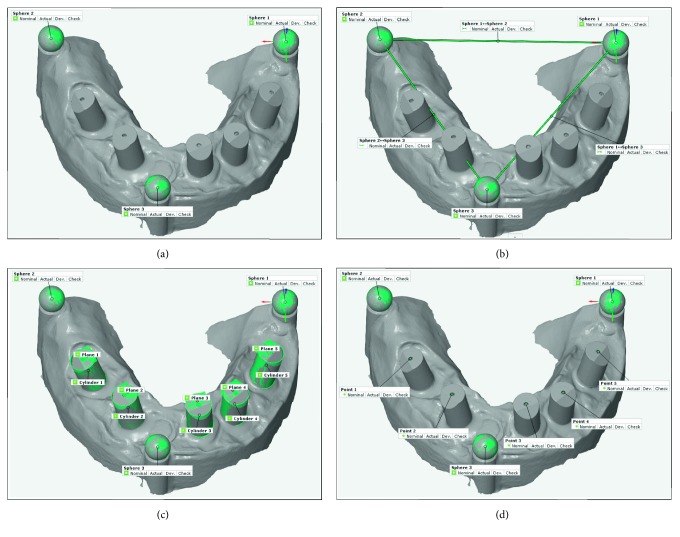
Four different measurement procedures. (a) Construction of the spheres and alignment of 3D model in the same CMM coordinate position. (b) Linear distances in between the center of the spheres. (c) Construction of planes and cylinders for all implants. (d) Installation of the center points of all implants.

A total of 120 scans were generated from the Atos and three IOS systems (30 virtual 3D models of the Atos and 30 virtual 3D models of each intraoral scanner). All 3D models were converted to standard tessellation language (STL) file format. Subsequently, each virtual 3D model was individually imported and measured once in a reverse engineering software program (GOM inspect software 2016, Rev. 95488). All scans were measured in the exact same way. The initial set after the virtual model had been imported into the software was the construction of the fitting element of the three spheres and then aligned the 3D model into the same *x*, *y*, and *z* CMM coordinate position (Figure 4(a)). Sphere 1 was used as anchorage or reference point. The linear distances between the center point of sphere 1 to sphere 2 (D1_D2), sphere 1 to sphere 3 (D1_D3), and sphere 2 to sphere 3 (D2_D3) were constructed and measured (Figure 4(b)). The distance between the spheres described the interarch distance between the posterior right, left, and anterior quadrants of the 3D model.

In order to measure the position and direction of all implants to reference sphere 1, the center point of each implant was located.  Figure 4(c) illustrates the construction of fitting cylinders and fitting planes. The best Gaussian fit was utilized as the fitting algorithm for the cylinders and planes. The center point of each implant was installed by constructing the intersecting point between cylinders and planes (Figure 4(d)). The center point of each implant was measured in the *x*, y, and *z* axes of the space. The *z*-axis represented the vertical direction of each implant. The *x* and *y* axes described the horizontal orientation of each implant.

### 2.6. Measurement Parameters

The measurement of each virtual 3D model consisted of the five implant center points in the *x*, *y*, and *z* axes, and three linear distances between the center of the spheres. One virtual 3D model was divided into a total of 18 parameters. Each scanner system resulted in 540 parameters. In total, 2160 measurement parameters were a result from the Atos and the three dental scanner groups. All measurements of the 3D models were conducted by one operator.

### 2.7. Datasets and Statistical Analysis

The measurement datasets were exported to SPSS (IBM SPSS Statistics version 25) where calculations for mean, 95% confidence interval, standard deviation (precision) of all scanners, and the difference between the mean values of all scanners and the true value (trueness) were made. The Shapiro–Wilks test was used to examine the normal distribution. One-way ANOVA was carried out to test for the hypothesis (*P* < 0.05). Furthermore, the post hoc test using least significant difference (LSD) was carried out to identify difference in implants and interarch distance between two specific intraoral scanners at 0.05 significant level.

## 3. Results

**Figure 5:**
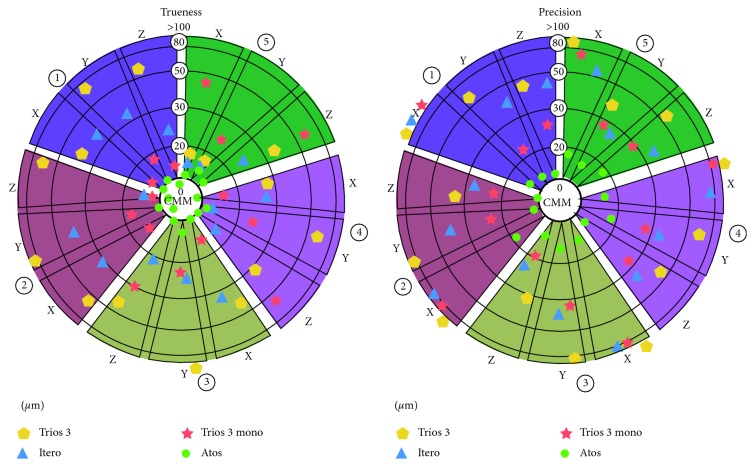
Bull's eyes chart showing the deviations between the mean of repeated measurements for all implant axes of all scanner devices and the true values (trueness) and the deviations between the results of independent measurements obtained within one scanner device under specific condition (precision). This chart is color-coded; each color represents the implant number (1–5) in the master model.  *μ*m = micron.

In this study, normal distribution was found by Shapiro–Wilks test in all intraoral scanners except the Atos scanner. A summary of results of all measurements for precision, trueness, mean, and 95% confidence interval per group is presented in Tables 2–5 and Figure 5. The trueness displayed positive and negative values, depending on whether the mean for Trios 3, Trios 3 mono, Itero element, and Atos were above or below the true value.

### 3.1. Trueness of Dental Scanners and Atos Scanner

#### 3.1.1. Trios 3

In the *y*-axis, Trios 3 showed least trueness when compared to the CMM value at ≤114 *μ*m. In the *z*-axis, it had the highest deviation when compared to the CMM dimension at ≤76 *μ*m. In the *x*-axis, it showed a trueness of ≤45 *μ*m. With regard to interarch distance, D1_3 showed the highest deviation in comparison with true dimension at 68 *μ*m.

#### 3.1.2. Trios 3 Mono

In the *y*-axis, Trios 3 mono showed an overall trueness of ≤27 *μ*m for all implants. In the *z*-axis, Trios 3 mono showed trueness when compared to the CMM dimension at ≤63 *μ*m. In the *x*-axis, Trios 3 mono displayed trueness at ≤47 *μ*m. The interarch measurements displayed an overall deviation of ≤45 *μ*m.

#### 3.1.3. Itero Element

In the *y*-axis, Itero presented trueness at ≤−41 *μ*m. In the *z*-axis, Itero showed an overall trueness of ≤25 *μ*m for all implants when compared to the CMM value. In the *x*-axis, Itero displayed deviation at ≤37 *μ*m. The interarch measurements displayed an overall deviation of ≤40 *μ*m.

#### 3.1.4. Atos Scanner

In the *y*-axis, it also showed highest trueness at ≤−8 *μ*m for all implants. In the *z*-axis, the Atos scanner displayed highest trueness at ≤3 *μ*m for all implants when compared to the true value. In the *x*-axis, it had highest trueness at ≤−2 *μ*m. The interarch measurements for Atos showed highest trueness of ≤−3 *μ*m.

### 3.2. Precision of Dental Scanners and Atos Scanner

#### 3.2.1. Trios 3

In the *x*-axis, Trios 3 had lowest precisions of ≤135 *μ*m and for the *y*-axis at ≤90 *μ*m. In the *z*-axis, Trios 3 showed a precision of ≤61 *μ*m. In the interarch measurements, Trios 3 displayed a precision of 46 *μ*m for D1_3 and 57 *μ*m for D2_3, while D1_2 had a lowest precision of ≤206 *μ*m.

#### 3.2.2. Trios 3 Mono

In the *z*-axis, Trios 3 mono showed an overall precision of ≤28 *μ*m and for the *y*-axis ≤33 *μ*m. However, *x*-axis showed a low precision of ≤100 *μ*m. In the interarch measurements, Trios 3 mono displayed a precision of 52 *μ*m for D1_3 and 22 *μ*m for D2_3. But, D1_2 presented a low precision of ≤111 *μ*m.

#### 3.2.3. Itero Element

In the *z*-axis, Itero showed an overall precision of ≤40 *μ*m and for the *y*-axis ≤44 *μ*m. However, *x*-axis showed a low precision of ≤101 *μ*m. In the interarch measurements, Itero displayed a precision of 28 *μ*m for D1_3 and 40 *μ*m for D2_3. On the other hand, D1_2 was had precision of ≤124 *μ*m.

#### 3.2.4. Atos Scanner

Atos scanner displayed a highest precision of ≤19 *μ*m for all implant parameters. Atos had a precision of ≤26 *μ*m in all interarch measurements.

### 3.3. Results of Statistical Analysis between CMM, Atos, and the Dental Scanner Systems

According to the results of the ANOVA, significant differences were found between the mean of all IOS devices and in comparison with the true value for all implant points and interarch distances, except for implant 1 *x*-axis (*p*=0.175), implant 4 *x*-axis (*p*=0.128), and D1_D2 (*p*=0.332). Table 5 displays the statistically significant difference in implants and interarch distances between two specific test groups.

## 4. Discussion

The aim of this study was to measure trueness and precision of different IOS systems when digitizing a fully edentulous cast with multiple implants. The results do not support the null hypothesis as statistically significant differences were found between the IOS devices in comparison with the true value. This in vitro study found variations in trueness and precision depending on the optical scanner systems utilized. On the basis of the *x*, *y*, and *z* parameters, Atos showed higher trueness and precision than all tested scanners. It would be preferable for the Atos system to be utilized by dentists; however, if the same scanning technology utilized by Atos was applied inside an intraoral device, the size of the hand-piece tip would be enormous and probably difficult to use intraorally. The authors of this study decided to use the Atos scanner as a reference scanner.

The material of the object surface being measured has an important influence on the scan result. The master model utilized in the current study consisted of stone material for the edentulous areas and tungsten metal for the implant positions (Figure 2). The stable metal part limits the movement or displacement of implant analogs in the master model to some extent and minimizes the risk of changes that could influence the results. However, the metallic parts could cause light reflective problems during the scanning process. Therefore, stone material was applied in the edentulous areas. The master model contained three spheres and five cylindrical scanning abutments, geometrical shapes that were used as fixed landmarks to assist the operator during scanning, making it possible to align and measure the virtual 3D model in a reliable way. In addition, the PEEK scanning abutment was selected because of its optical properties that produce good light dispersion.

The correlation between trueness and precision is a significant aspect in choosing a suitable IOS scanner for the intended application. The data that resulted from the current study on implant measurements for Trios 3 showed trueness data ≤114 *μ*m and Trios 3 mono presented trueness data ≤63 *μ*m. The same data from the Itero element showed trueness values ≤−41 *μ*m. There is no consensus between studies concerning the standard precision level for each intraoral scanner system. In this study, the reference Atos showed a precision of ≤19 *μ*m in all implant dimensions. On the other hand, Trios 3 mono and Itero element presented precision of approximately <45 *μ*m in the *y* and *z* direction, but the *x*-axis displayed low precision at ≤101 *μ*m. Also, Trios 3 has the lowest precision of ≤135 *μ*m in the *x*-axis. The precision of those IOS systems was not optimal enough to produce several virtual models that are closely comparable to each other in the *x*-axis. Thus, in the present study, the results from precision indicated that all tested IOS systems were not reliable enough for scanning fully edentulous arch with multiple implants.

The comparison of obtained values with the available literature is difficult; to our knowledge, there is no study using an identical measuring method to that of the present study. Some studies have utilized the best fit alignment method to assess direct digital impression of dentate arch or edentulous arch with multiple implants [4, 7, 8, 16]. The method refers to superimposing tested scans onto a reference scan of a physical model in different 3D software. The protocol can generate errors caused by alignment computing processes because of the deviations between the superimposed areas, especially in larger datasets such as full-arch scans [17, 18]. However, some studies have assessed accuracy of IOS devices by measuring only the horizontal linear distances between the reference landmarks [6, 9, 15, 19, 20]. The use of different IOS versions in the majority of the studies had an impact on the dissimilar results obtained. It can be assumed that the different materials and methods can lead to conflicting results in relation to the accuracy of IOS systems.

Intraoral scanners do not have the ability to scan the entire arch in one image. The small hand-piece unit has to move across the arch. The scanner's software stitches the images of the arch and implant and combines one image after another image; this seems to induce errors. The clear effect of the stitching processes producing errors proportional to the scan distance, as noted in this study, has also been documented in other studies [6, 9, 10]. The results indicated that the errors were increased if the interarch distance between the spheres is horizontally enlarged or reduced in width. The data found from the current study on interarch measurements for Trios 3 showed lowest trueness among other groups, but all tested scanners had low precision. Contradictory results can be found in the study that measured the trueness and precision of Trios (unclear version) and Itero (unknown version) on scanning dental arch [19]. Muallah and coworkers utilized five boreholes on their 3D printed master cast that were measured utilizing tactile CMM, and the cast was scanned thirty-two times per IOS device. Two of the boreholes replaced the first molars; the intermolar width (IMW) measurement in that study is similar to the interarch measurement D1_D2 in the current study. They found that Trios and Itero had better trueness and precision values. The difference in the results could be caused by different model materials, a different 3D analyzing program, a different software version of the scanners, and different reference landmark size and shapes. The most significant difference that led to better results in their study was that the arch is not edentulous. It should be noted that the IOS software can cause poor stitching and matching procedure of the single point clouds during the scanning process of the edentulous area. By improving the matching algorithms, some IOS devices could attain digital impression of fully edentulous arches with multiple implants cases in the near future.

The accuracy of full-arch scan is correlated with the correct scanning strategy. Muller et al. [13] reported that the zigzag strategy has a lower trueness value but a better precision value than buccal-occlusal-palatal (BOP) strategy. In the present study, both Trios scanners used the BOP strategy and Itero element used the zigzag strategy to scan the master model. The results showed that the Itero element presented better trueness and precision outcomes than Trios 3, and statistically significant difference was found between them. However, Trios 3 mono and Itero element presented comparable results, with a statistically significant difference. Trios 3 mono has no color feature, which might explain why the scanning strategy played no significant role between the two different systems. It can be suggested that the color software collects information related to the color of the object being scanned during the scanning process. Subsequently, color software may interfere with the quality of the stitching process. Lastly, this study failed to account for saliva, soft tissue isolation, patient movement, and humidity in the oral environment. These patient variables may affect accuracy considerably in a clinical situation. The laboratory procedures after scanning also introduce errors that need to be considered.

No power calculation was performed in this study, which could be considered a limitation. The sample size was instead based on ISO standard 12836:2015 which states that, to test a digital device in terms of accuracy, trueness, and precision, the measurement has to be repeated 30 times, which is what was done in this paper [2]. Another limitation of this present study was that all tested scanner systems have similar scanning technology.

The findings in this study do not relate directly to the definitive prostheses. However, the level of model-less digital production tolerances for full-arch framework supported by teeth or implants is not clearly known yet. One of the most common treatment modalities is an implant-supported fixed full-arch framework. Örtorp et al. [20] reported that the production tolerance for implant-supported fixed prostheses should be in the range of 20–100 *μ*m tolerance. Comparison of their findings with this current study results should be done with consideration since the frameworks in their study were produced from scanning the implant master cast with a laboratory scanner. One good example is that the trueness of Trios 3 was ≤114 *μ*m at *y*-axis with precision 135 *μ*m at *x*-axis. This could generate a misfit between the components of the whole implant prosthesis, or it could produce unfit implant frameworks. A pilot clinical study investigated the digital impression of 25 patients with two implants in the mandible and reported that errors were too large to fabricate well-fitting implant frameworks [5].

The results of this present study provide the knowledge of the nature of deviation in full-arch digital impression and can help to avoid these errors in future. The study was conducted to provide knowledge for dental professionals to understand and control the digital scanning process. Future studies need to include more dental scanner systems and compare them in different clinical scenarios. The whole model-less digital production for full-tooth-fixed supported prostheses and full implant-fixed supported prostheses workflow should be assessed.

## 5. Conclusion

Within the limitations of this in vitro study, the results suggest significant differences between IOS devices when scanning fully edentulous arch with multiple implants. The main observation was the low precision for all intraoral scanners, suggesting that the intraoral scanning devices are unreliable for scanning fully edentulous arch with multiple implants. Two scanners, however, Trios 3 mono and Itero element showed fair trueness.

## Figures and Tables

**Table 1:** Information about the intraoral scanner systems.

System	Manufacturer	Software	Scanning technology	Scan protocol	Acquisition	Powder application	Export
Trios 3	3shape	1.4.5.1	Conofocal microscopy	BOP	Video sequence	No	STL
Trios 3 mono	3shape	1.4.6.4	Conofocal microscopy	BOP	Video sequence	No	STL
Itero element	Cadent Inc.	1.4.0.318	Conofocal microscopy	Zig-zag	Video sequence	No	STL

B = buccal; O = occlusal; P = palatal.

**Table 2:** Results of precision and trueness of all implants and interarch distances.

Parameters (mm)	n	Atos		Trios 3		Trios 3 mono		Itero elements	
		Precision	Trueness	Precision	Trueness	Precision	Trueness	Precision	Trueness
Implant 1 X	30	0.009	−0.001	0.135	0.042	0.100	−0.009	0.101	0.037
Implant 1 Y	30	0.004	−0.004	0.073	0.079	0.023	0.016	0.038	−0.034
Implant 1 Z	30	0.002	0.000	0.047	0.063	0.025	−0.006	0.040	0.025

Implant 2 X	30	0.018	0.000	0.127	0.054	0.095	−0.014	0.083	0.032
Implant 2 Y	30	0.006	−0.004	0.094	0.095	0.027	0.018	0.044	−0.041
Implant 2 Z	30	0.002	0.000	0.035	0.076	0.024	−0.003	0.029	0.015

Implant 3 X	30	0.014	−0.001	0.133	−0.047	0.097	0.015	0.081	0.032
Implant 3 Y	30	0.014	−0.007	0.097	0.114	0.033	−0.025	0.041	−0.025
Implant 3 Z	30	0.007	0.002	0.033	0.046	0.019	−0.025	0.025	0.021

Implant 4 X	30	0.016	0.000	0.129	−0.029	0.082	0.015	0.075	0.028
Implant 4 Y	30	0.019	−0.003	0.077	0.066	0.029	−0.027	0.034	−0.007
Implant 4 Z	30	0.008	0.001	0.049	0.031	0.027	0.056	0.039	0.015

Implant 5 X	30	0.018	−0.002	0.099	−0.018	0.059	0.047	0.055	0.010
Implant 5 Y	30	0.014	−0.008	0.039	0.018	0.026	−0.025	0.026	−0.012
Implant 5 Z	30	0.018	0.003	0.063	0.035	0.028	0.063	0.033	0.025

D1_D2	30	0.005	−0.003	0.206	0.003	0.111	−0.034	0.124	0.040
D1_D3	30	0.011	−0.001	0.046	0.068	0.052	−0.017	0.028	−0.009
D2_D3	30	0.026	−0.002	0.061	0.014	0.022	0.045	0.040	−0.033

*n =* number of scans; mm = millimeter; *n* = number; D1_D2 = distance from sphere 1 to sphere 2; D1_D3 = distance from sphere 1 to sphere 3; D2_D3 = distance from sphere 2 to sphere 3.

**Table 3:** Results of mean and 95% confidence interval for the mean of all implants and interarch distances.

Parameters (mm)	n	Atos			Trios 3			Trios 3 Mono			Itero elements		
			95% confidence interval for mean			95% confidence interval for mean			95% confidence interval for mean			95% confidence interval for mean	
		Mean	Lower bound	Upper bound	Mean	Lower bound	Upper bound	Mean	Lower bound	Upper bound	Mean	Lower bound	Upper bound
Implant 1 X	30	47.016	47.013	47.020	47.060	47.010	47.111	47.009	46.972	47.046	47.055	47.017	47.093
Implant 1 Y	30	19.870	19.869	19.872	19.953	19.926	19.980	19.891	19.882	19.899	19.840	19.826	19.854
Implant 1 Z	30	4.361	4.360	4.362	4.424	4.407	4.442	4.356	4.347	4.365	4.386	4.371	4.401

Implant 2 X	30	37.597	37.590	37.604	37.652	37.604	37.699	37.583	37.548	37.619	37.629	37.598	37.660
Implant 2 Y	30	31.030	31.028	31.032	31.129	31.093	31.164	31.052	31.042	31.063	30.993	30.977	31.009
Implant 2 Z	30	6.147	6.146	6.148	6.223	6.210	6.236	6.144	6.135	6.153	6.162	6.152	6.173

Implant 3 X	30	20.618	20.613	20.623	20.571	20.521	20.621	20.633	20.597	20.669	20.650	20.620	20.681
Implant 3 Y	30	35.667	35.662	35.672	35.788	35.752	35.824	35.649	35.637	35.661	35.649	35.634	35.664
Implant 3 Z	30	9.087	9.085	9.090	9.131	9.119	9.143	9.115	9.108	9.122	9.106	9.097	9.116

Implant 4 X	30	11.911	11.906	11.917	11.883	11.835	11.931	11.927	11.896	11.957	11.940	11.912	11.968
Implant 4 Y	30	30.751	30.743	30.758	30.820	30.791	30.849	30.727	30.716	30.738	30.747	30.734	30.759
Implant 4 Z	30	6.903	6.900	6.907	6.934	6.916	6.952	6.958	6.948	6.968	6.918	6.903	6.932

Implant 5 X	30	3.633	3.627	3.640	3.617	3.580	3.654	3.682	3.660	3.704	3.645	3.625	3.665
Implant 5 Y	30	18.106	18.101	18.112	18.133	18.118	18.147	18.090	18.080	18.099	18.103	18.093	18.113
Implant 5 Z	30	5.535	5.528	5.542	5.567	5.544	5.590	5.596	5.585	5.606	5.558	5.545	5.570

D1_D2	30	53.687	53.685	53.688	53.692	53.615	53.769	53.655	53.614	53.697	53.729	53.682	53.775
D1_D3	30	50.923	50.919	50.928	50.990	50.975	51.009	50.908	50.888	50.927	50.915	50.905	50.926
D2_D3	30	47.608	47.599	47.618	47.624	47.601	47.647	47.655	47.647	47.663	47.578	47.563	47.592

mm = millimeter; *n* = number; D1_D2 = distance from sphere 1 to sphere 2; D1_D3 = distance from sphere 1 to sphere 3; D2_D3 = distance from sphere 2 to sphere 3.

**Table 4:** The results of one-way ANOVA between all groups in comparison with the true value for implants and interarch distances.

ANOVA					
	Sum of squares	df	Mean square	F	Sig.
Implant 1 X	0.062	4	0.016	1.614	0.175
Implant 1 Y	0.206	4	0.051	28.215	0.000
Implant 1 Z	0.088	4	0.022	19.774	0.000

Implant 2 X	0.086	4	0.021	2.654	0.037
Implant 2 Y	0.294	4	0.074	25.429	0.000
Implant 2 Z	0.122	4	0.030	46.979	0.000

Implant 3 X	0.104	4	0.026	3.057	0.020
Implant 3 Y	0.404	4	0.101	32.890	0.000
Implant 3 Z	0.030	4	0.008	14.418	0.000

Implant 4 X	0.053	4	0.013	1.827	0.128
Implant 4 Y	0.149	4	0.037	17.883	0.000
Implant 4 Z	0.051	4	0.013	10.865	0.000

Implant 5 X	0.069	4	0.017	4.176	0.003
Implant 5 Y	0.029	4	0.007	9.501	0.000
Implant 5 Z	0.058	4	0.014	9.420	0.000

D1_D2	0.081	4	0.020	1.160	0.332
D1_D3	0.135	4	0.034	23.715	0.000
D2_D3	0.094	4	0.024	14.637	0.000

D1_D2 = distance from sphere 1 to sphere 2; D1_D3 = distance from sphere 1 to sphere 3; D2_D3 = distance from sphere 2 to sphere 3; df = degree of freedom; F = analysis of variance; Sig. = significant.

**Table 5:** The result of post hoc test between the intraoral scanners in implants and interarch distances.

	Trios 3 vs. mono	Trios 3 vs. Itero	Mono vs. Itero Sig.
	Sig.	Sig.	
Implant 1 X	0.045	0.841	0.071
Implant 1 Y	0.000	0.000	0.000
Implant 1 Z	0.000	0.000	0.001

Implant 2 X	0.004	0.334	0.052
Implant 2 Y	0.000	0.000	0.000
Implant 2 Z	0.000	0.000	0.006

Implant 3 X	0.011	0.001	0.467
Implant 3 Y	0.000	0.000	0.991
Implant 3 Z	0.007	0.000	0.154

Implant 4 X	0.052	0.011	0.548
Implant 4 Y	0.000	0.000	0.096
Implant 4 Z	0.006	0.069	0.000

Implant 5 X	0.000	0.069	0.027
Implant 5 Y	0.000	0000	0.068
Implant 5 Z	0.005	0.364	0.000

D1_D2	0.282	0.29	0.034
D1_D3	0.000	0.000	0.439
D2_D3	0.003	0.000	0.000

D1_D2 = distance from sphere 1 to sphere 2; D1_D3 = distance from sphere 1 to sphere 3; D2_D3 = distance from sphere 2 to sphere 3; vs. = versus; mono = Trios 3 mono; Sig. = significant.

## Data Availability

The data used to support the findings of this study are available from the corresponding author upon reasonable request.

## References

[1] Mörmann W. H. (2006). The evolution of the CEREC system. *Journal of the American Dental Association*.

[2] ISO 12836 (2015). *Dentistry–Digitizing Devices for CAD/CAM Systems for Indirect Restorations–Test for Methods for Assessing Accuracy*.

[3] Nedelcu R., Olsson P., Nystrom I., Ryden J., Thor A. (2018). Accuracy and precision of 3 intraoral scanners and accuracy of conventional impressions: a novel in vivo analysis method. *Journal of Dentistry*.

[4] Imburgia M., Logozzo S., Hauschild U., Veronesi G., Mangano C., Mangano F. G. (2017). Accuracy of four intraoral scanners in oral implantology: a comparative in vitro study. *BMC Oral Health*.

[5] Andriessen F. S., Rijkens D. R., van der Meer W. J., Wismeijer D. W. (2014). Applicability and accuracy of an intraoral scanner for scanning multiple implants in edentulous mandibles: a pilot study. *Journal of Prosthetic Dentistry*.

[6] Van der Meer, Wicher J., Andriessen F. S., Daniel W., Ren Y. (2012). Application of intra-oral dental scanners in the digital workflow of implantology. *PLoS One*.

[7] Ender A., Mehl A. (2015). In-vitro evaluation of the accuracy of conventional and digital methods of obtaining full-arch dental impressions. *Quintessence International*.

[8] Mangano F. G., Veronesi G., Hauschild U., Mijiritsky E., Mangano C. (2016). Trueness and precision of four intraoral scanners in oral implantology: a comparative in vitro study. *PloS One*.

[9] Giménez B., Özcan M., Martínez-Rus F., Pradíes G. (2014). Accuracy of a digital impression system based on parallel confocal laser technology for implants with consideration of operator experience and implant angulation and depth. *International Journal of Oral and Maxillofacial Implants*.

[10] Giménez B., Hassan B., Özcan M., Pradíes G. (2017). An in vitro study of factors influencing the performance of digital intraoral impressions operating on active wavefront sampling technology with multiple implants in the edentulous maxilla. *Journal of Prosthodontics*.

[11] Flügge T. V., Schlager S., Nelson K., Nahles S., Metzger M. C. (2013). Precision of intraoral digital dental impressions with iTero and extraoral digitization with the iTero and a model scanner. *American Journal of Orthodontics and Dentofacial Orthopedics*.

[12] Kim J. H., Kim K. B., Kim S. H., Kim W. C., Kim H. Y., Kim J. H. (2015). Quantitative evaluation of common errors in digital impression obtained by using an LED blue light in-office CAD/CAM system. *Quintessence International*.

[13] Müller P., Ender A., Joda T., Katsoulis J. (2016). Impact of digital intraoral scan strategies on the impression accuracy using the TRIOS Pod scanner. *Quintessence International*.

[14] Ender A., Mehl A. (2013). Influence of scanning strategies on the accuracy of digital intraoral scanning systems. *International Journal of Computerized Dentistry*.

[15] Flügge T. V., Att W., Metzger M. C., Nelson K. (2016). Precision of dental implant digitization using intraoral scanners. *International Journal of Prosthodontics*.

[16] Papaspyridakos P., Gallucci G. O., Chen C. -J., Hanssen S., Naert I., Vandenberghe B. (2016). Digital versus conventional implant impressions for edentulous patients: accuracy outcomes. *Clinical Oral Implants Research*.

[17] Güth J. F., Edelhoff D., Schweiger J., Keul C. (2016). A new method for the evaluation of the accuracy of full-arch digital impressions in vitro. *Clinical Oral Investigations*.

[18] Kuhr F., Schmidt A., Rehmann P., Wostmann B. (2016). A new method for assessing the accuracy of full arch impressions in patients. *Journal of Dentistry*.

[19] Muallah J., Wesemann C., Nowak R. (2017). Accuracy of full-arch scans using intraoral and extraoral scanners: an in vitro study using a new method of evaluation. *International Journal of Computerized Dentistry*.

[20] Örtorp A., Jemt T., Bäck T., Jalevik T. (2003). Comparisons of precision of fit between cast and CNC-milled titanium implant frameworks for the edentulous mandible. *International Journal of Prosthodontics*.

